# Teledermatology Diagnostic Accuracy: A Randomized Cohort Study Comparing Three Image Acquisition Techniques

**DOI:** 10.1155/ijta/5789165

**Published:** 2025-09-24

**Authors:** Serena Saade, Dana Maria Khoury, William Abou Shahla, Carla Stephan, Nicole Charbel, Ronny Joukhdar, Martine El Bejjani, Mira Bekdache, Sara Mansour, Dana Saade

**Affiliations:** ^1^Department of Dermatology, American University of Beirut Medical Center, Beirut, Lebanon; ^2^Clinical Research Institute, Faculty of Medicine, American University of Beirut, Beirut, Lebanon

**Keywords:** diagnostic accuracy, image acquisition, skin diseases, teledermatology

## Abstract

Teledermatology is increasingly recognized as a valuable tool for expanding access to dermatologic care, particularly in underserved and remote settings. This randomized prospective cohort study, the first of its kind in the region, examined the diagnostic and treatment concordance of teledermatologists compared to in-clinic dermatologists, with a focus on the impact of image acquisition methods. A total of 360 adult patients presenting for dermatologic evaluation at the American University of Beirut Medical Center were enrolled. Each patient contributed three sets of skin lesion photographs: unassisted patient-taken, assisted patient-taken after brief standardized training, and resident-taken under clinical conditions. Using a store-and-forward model, teledermatologists reviewed one randomized image set per patient. Their diagnoses and treatment decisions were compared to in-person assessments, which served as the reference standard. Diagnostic concordance improved with image quality: 79% for unassisted patient images, 84% for assisted images, and 87% for resident-taken images. Treatment concordance similarly increased from 38% to 44% to 45%, while the need to request additional history declined from 64% to 47%. Acne showed the highest diagnostic match across all image types. These findings support the clinical validity of store-and-forward teledermatology and highlight the value of patient education in improving image quality and diagnostic outcomes. As the first regional study to systematically evaluate real-world image acquisition methods, it offers a practical roadmap for integrating teledermatology into routine dermatologic care.

## 1. Introduction

The field of dermatology primarily relies on visual inspection for diagnosis [1]. However, in many cases, additional information—such as the history of present illness, past medical history, physical examination, and confirmatory testing like blood work or skin biopsies—is often required for accurate clinical assessment. With the rapid expansion of digital health platforms, remote dermatologic consultations have become increasingly common. The COVID-19 pandemic accelerated this shift, establishing teledermatology as a safe and effective alternative during periods of restricted in-person access.

Teledermatology, defined as the remote delivery of dermatologic care [2], typically takes two forms: asynchronous “store-and-forward” consultations, in which images are captured and reviewed at a later time, and synchronous live video interactions between clinician and patient [3]. While face-to-face initial encounters remain the diagnostic gold standard, teledermatology offers a valuable means of overcoming barriers to access. According to a systematic review, teledermatology has shown itself to be a useful and convenient tool for the management of common ambulatory dermatoses, such as eczema and acne, particularly during the COVID-19 pandemic [2]. Moreover, in regions with limited access to healthcare, teledermatology holds promise as a tool to bridge critical care gaps.

Despite its growing use, most studies on teledermatology rely on clinician-captured images, which may not reflect real-world conditions. In routine care, patients are often responsible for photographing their own lesions, frequently without guidance, raising concerns about image quality and diagnostic reliability.

This study is aimed at evaluating the diagnostic accuracy of dermatologists in identifying skin diseases using smartphone-acquired photographs. Using a store-and-forward model, we assessed three image acquisition methods: patient-taken without guidance, patient-taken after brief training, and resident-taken photographs. By comparing teledermatology assessments against in-clinic evaluations, we aim to determine the impact of patient training and image quality on diagnostic concordance, with the goal of informing more effective integration of teledermatology into clinical practice.

## 2. Materials and Methods

This was a single-center, randomized prospective cohort study conducted at the American University of Beirut Medical Center (AUBMC), a major tertiary referral hospital in Lebanon and the Middle East. The study protocol was reviewed and approved by the Institutional Review Board of the American University of Beirut (IRB Approval Number: BIO-2020-0241), and all procedures were conducted in accordance with the Declaration of Helsinki and relevant institutional guidelines. Written informed consent was obtained from all participants prior to enrollment.

### 2.1. Study Setting and Participants

Eligible participants were adults (≥ 18 years of age) presenting for an initial dermatologic consultation at AUBMC's outpatient department or private dermatology clinics. Patients were excluded if they had already started treatment for their condition for more than 1 week or if in-clinic dermatologists could not reach a consensus on diagnosis.

All eligible patients were privately consented for participation. After clinical examination by an attending dermatologist, patients were independently evaluated by a second attending dermatologist and a dermatology resident for diagnostic confirmation. The consensus diagnosis among all three in-clinic evaluators was considered the reference standard (“true diagnosis”).

### 2.2. Image Acquisition and Study Groups

Each participant underwent three sequential forms of skin lesion photography:

1. *Unassisted patient-taken photographs*: Patients captured their own images without any instruction using personal smartphones.
2. *Assisted patient-taken photographs*: Patients were provided with brief, standardized training on photographic technique (Form S4), including guidance on lighting, focus, framing, and lesion visibility, and then recaptured their images.
3. *Resident-taken photographs*: A dermatology resident took standardized clinical photographs under controlled lighting.

Standardized images were taken by dermatology residents using an iPhone 13 Pro equipped with a 12 MP main camera. Photographs were captured in a well-lit dermatology examination room, using ambient daylight and ceiling-mounted LED lighting to ensure consistent illumination and minimize shadows or glare. Before each image, residents ensured adequate lighting and removed any distractors such as jewelry or clothing to maintain focus on the lesion. Whenever possible, the lesion was surrounded by a white background (e.g., gauze or paper) to improve contrast. Images were taken at a fixed zoom level of 1× from an approximate distance of 30 cm, with the camera held perpendicular to the skin surface. Residents tapped the screen to ensure proper focus and reviewed each image immediately; if a photo appeared blurry, it was retaken. For each case, two images were acquired: one from a distance to indicate the anatomic location of the lesion and one close-up to assess lesion details. A ruler was included for scale when applicable. Images were deidentified and securely stored using a store-and-forward teledermatology model. Identifying information was stored separately to maintain confidentiality.

### 2.3. Teledermatology Assessment

Participants' images were distributed to three separate groups of teledermatology reviewers (attending dermatologists and residents), with each group receiving only one image set per patient. No evaluator reviewed images or cases they had seen in clinic. For each image set, teledermatologists were asked to record the following in Form S3:

• Diagnosis and proposed treatment
• Time to reach diagnosis (in seconds)
• Whether patient history was required
• Confidence in diagnosis (rated on a scale from 1 to 10)
• Recommended follow-up interval (in days)

A total of 360 patients were enrolled in the study.

### 2.4. Statistical Analysis

The primary outcomes were diagnostic and treatment concordance between teledermatologists and in-clinic evaluations, stratified by image acquisition method. Secondary outcomes, as assessed by teledermatologists while reviewing the images, included time taken to establish a diagnosis, level of confidence in the diagnosis (rated on a 1–10 scale), recommended follow-up interval (in days), and whether the teledermatologist requested additional clinical history to make a diagnosis.

We used mixed-effects models with patient ID as a random effect to account for repeated measures across image types. Intraclass correlation coefficients (ICCs) were calculated to assess outcome clustering within patients. Descriptive statistics were reported for each modality, and differences in clinical outcomes were compared across image types and rater groups (attendings vs. residents). Subgroup analyses were conducted for acne/rosacea, papulosquamous disorders, and malignant neoplasms. Associations with patients' education level were also examined.

Interrater agreement was assessed using kappa coefficients and ICCs. Interaction terms were tested to evaluate whether rater expertise modified the relationship between image type and clinical outcomes. All analyses were conducted using Stata, with a significance level of *p* < 0.05 for primary analyses and *p* < 0.1 for interactions.

### 2.5. Preprint Disclosure

A preprint version of this study by Saade et al. was previously published on Research Square and is available at 10.21203/rs.3.rs-4968038/v1 [4]. The preprint has not undergone peer review.

## 3. Results

A total of 360 patients were included in the study, with an average age of 42.2 years (range: 18–85 years). Among them, 129 were male and 231 were female. Each participant contributed three sets of images for evaluation: (1) unassisted patient-taken photographs, (2) assisted patient-taken photographs following brief in-clinic training, and (3) resident-taken photographs captured under standardized clinical conditions.

In this study, diagnosis match was defined as agreement between the diagnosis made by the teledermatologist and the reference diagnosis established during the in-clinic visit. Similarly, treatment match referred to concordance between the treatment recommended by the teledermatologist and that prescribed in person by the dermatologist.

Table 1 summarizes clinical outcomes across the three image acquisition methods. The proportion of diagnosis matches improved progressively with image quality: 79% for unassisted patient-taken photographs, 84% for assisted patient-taken photographs, and 87% for resident-taken photographs. A similar upward trend was observed in treatment match rates, which increased from 38% in the unassisted group to 44% in the assisted group and 45% in the resident group.

When reviewing images, teledermatologists had the option to request the patient's medical history if needed for diagnosis. The frequency of such requests decreased as image quality improved, occurring in 64% of unassisted cases, 59% of assisted cases, and 47% of resident-taken photograph evaluations. In parallel, the average time taken by teledermatologists to reach a diagnosis declined across modes (18.79, 17.01, and 16.27 s, respectively), while the recommended follow-up interval increased (16.18, 18.87, and 20.39 days). Confidence in the diagnosis, rated on a scale from 1 to 10, also improved steadily (mean scores: 6.43, 6.73, and 6.91). Tables S1–S3 show diagnosis and treatment agreement and related outcomes across the three assessment modes for attendings and residents.

Table 2 presents diagnosis match rates stratified by major dermatologic categories. Acne and rosacea showed the highest diagnostic concordance across all image modes (95%, 96%, and 98%), followed by papulosquamous dermatoses (74%, 81%, and 83%) and malignant neoplasms (76%, 90%, and 86%). Among correctly diagnosed malignancies, biopsy was recommended by teledermatologists in 82% of both patient-taken photograph groups (unassisted and assisted) and in 92% of resident-taken photograph cases.

Table 3 presents the results of mixed-effects regression analyses. Compared to resident-taken photographs, unassisted patient-taken photographs were associated with significantly lower odds of diagnosis match (OR 0.59; 95% CI: 0.45–0.79) and treatment match (OR 0.76; 95% CI: 0.59–0.97). Teledermatologists were also nearly twice as likely to request medical history in these cases (OR 1.98; 95% CI: 1.55–2.82). Additionally, diagnoses took longer to reach (*β* = 2.51; 95% CI: 1.20–3.82), confidence in diagnosis was lower (*β* = −0.48; 95% CI: −0.70 to −0.26), and the recommended follow-up interval was shorter (*β* = −4.21; 95% CI: −7.45 to −0.97).

Assisted patient-taken photographs, on the other hand, demonstrated diagnostic and treatment match rates comparable to those of resident-taken photographs. The only significant difference observed was a greater likelihood of teledermatologists requesting patient history (OR 1.59; 95% CI: 1.25–2.03).

When comparing rater groups, residents had lower odds of diagnosis match (OR 0.66; 95% CI: 0.52–0.83) compared to attending dermatologists, but they were more likely to recommend the correct treatment (OR 1.43; 95% CI: 1.17–1.75) and more frequently requested patient history (OR 1.43; 95% CI: 1.17–1.75). Residents also reached diagnoses more quickly (*β* = −5.42; 95% CI: −6.49 to −4.35) but reported lower confidence in their assessments (*β* = −1.49; 95% CI: −1.66 to −1.31). Tables S4 and S5 detail diagnostic and treatment agreement across modes and raters.

Lastly, patient education level (university vs. nonuniversity) was not significantly associated with any clinical outcomes, including diagnosis match, treatment match, or teledermatologist decision-making (Table 4; all *p* > 0.05).

## 4. Discussion

Teledermatology represents a transformative advancement in healthcare delivery, especially for patients with limited access to in-person dermatologic care. Prior studies, such as one conducted in Amsterdam, have emphasized the utility of teledermoscopy in enhancing dermatologic care in general practice settings [5]. Building on this foundation, our randomized prospective cohort study further explores a critical and underexamined aspect of teledermatology: the influence of image acquisition method and patient training on diagnostic accuracy and treatment alignment.

Our research unfolds a progressive trend in diagnostic and treatment matching across three modes of image capture: unassisted patient-taken photographs, assisted patient-taken photographs, and resident-taken photographs. The correlation is evident—as the level of expertise and assistance in image capture increases, so does the precision of diagnoses and treatment plans. These findings highlight the critical role of image quality in teledermatologic accuracy and support the implementation of simple, scalable interventions such as brief patient training or clear instructional guidance to improve diagnostic reliability.

The introduction of brief educational tools, such as video tutorials or printed guides on optimal image capture, aligns with existing teledermatology guidelines and offers a practical, low-cost strategy to improve image quality across diverse patient populations [6]. By standardizing photographic technique, these tools could serve as valuable adjuncts in remote dermatologic consultations and reduce variability introduced by patient-captured images.

Our study also reveals condition-specific variability in diagnostic concordance. While acne cases exhibited consistently high diagnostic matching across all modes, more complex disorders like malignant neoplasms and papulosquamous disorders showcased variations, emphasizing the need for careful consideration of image quality and context. Notably, resident-taken photographs achieved the highest diagnostic accuracy for malignant lesions, a finding with meaningful clinical implications. The improved diagnostic match in malignancy cases with better quality images suggests a reduced likelihood of misdiagnosis or delayed treatment for potentially serious conditions.

These results are consistent with prior studies highlighting teledermatology's role in the early detection of skin cancers, particularly melanoma. Several investigations have reported strong concordance between face-to-face and remote evaluations in high-risk populations [7, 8]. However, diagnostic accuracy for pigmented lesions remains mixed, with lower agreement reported in atypical nevi, lentigines, and early melanomas. These inconsistencies reaffirm the importance of high-quality image acquisition and reinforce the need for cautious interpretation in complex cases [7, 8].

Our results also shed light on the performance of teledermatology evaluators. Residents demonstrated higher odds of recommending appropriate treatment and of requesting clinical history but were less likely to match the correct diagnosis compared to attending dermatologists. This may reflect a more cautious or algorithmic approach among trainees, highlighting the need for targeted training in image-based diagnostic reasoning.

Importantly, we found no significant association between patient education level and clinical outcomes, suggesting that teledermatology can be equitably applied across diverse sociodemographic groups. This reinforces the utility of the “store and forward” model in low-resource settings and supports its integration into broader public health strategies.

While teledermatology presents substantial advantages, it comes with inherent limitations. The inability to conduct a comprehensive full-body examination, potential legal risks associated with diagnostic errors, and the risk of overlooking other skin lesions while focusing on a specific one are noteworthy concerns [5, 9]. Additionally, our study used in-clinic expert consensus as the reference standard rather than histopathologic confirmation. Although this approach reflects real-world practice, it introduces potential variability, especially in complex or ambiguous presentations. Furthermore, the inclusion of a broad range of dermatologic conditions, while increasing generalizability, may limit condition-specific conclusions and suggest the need for larger, stratified studies.

Our study acknowledges specific limitations, including the reliance on in-person expert opinions as the sole basis for the “true diagnosis” due to constraints on pathological confirmation. Diverse management strategies and intervariability among experts further contribute to differences in diagnostic and treatment approaches. The study's broad recruitment strategy adds complexity, necessitating larger scale studies to effectively categorize the utility of teledermatology for specific conditions.

This is the first study in the region to systematically compare diagnostic accuracy across three distinct image acquisition methods: unassisted patient-taken photographs, assisted patient-taken photographs, and clinician-taken photographs within a randomized prospective teledermatology framework. Our findings underscore the transformative potential of teledermatology to expand access and improve care while highlighting the pivotal impact of image quality on diagnostic precision. By demonstrating that even brief patient education can significantly enhance diagnostic outcomes, this study offers a practical roadmap for integrating teledermatology more effectively into routine clinical practice. Its future success will depend not only on the use of digital platforms but also on empowering both patients and clinicians through training, standardization, and thoughtful implementation.

## Figures and Tables

**Table 1:** Match in diagnosis and treatment and related outcomes across the three assessment modes.

	**Pictures taken by patient without assistance**		**Pictures taken by patient with assistance**		**Pictures taken by resident**
	**Mean** ^**a**^	**95% CI**	**Mean** ^**a**^	**95% CI**	**Mean** ^**a**^	**95% CI**
Diagnosis match^b^	0.79	0.75, 0.84	0.84	0.80, 0.88	0.87	0.83, 0.90
Treatment match^b^	0.38	0.33, 0.43	0.44	0.38, 0.50	0.45	0.39, 0.50
Asked for history	0.64	0.59, 0.69	0.59	0.53, 0.64	0.47	0.42, 0.52
Mean follow-up (in days)	16.18	13.41, 18.96	18.87	16.09, 21.65	20.39	17.62, 23.17
Time to diagnosis (in seconds)	18.79	17.61, 19.96	17.01	15.83, 18.18	16.27	15.10, 17.45
Confidence	6.43	6.23, 6.63	6.73	6.53, 6.93	6.91	6.71, 7.11

^a^Means represent margin values estimated using mixed models accounting for repeated assessments across modes (i.e., repeated patient subjects), with modes of evaluation and rater as predictors.

^b^Means of percent matches (ranging from 0 to 1).

**Table 2:** Diagnosis and treatment match for specific conditions across the three assessment modes.

	**Pictures taken by patient without assistance**		**Pictures taken by patient with assistance**		**Pictures taken by resident**	
	**Mean** ^**a**^	**95% CI**	**Mean** ^**a**^	**95% CI**	**Mean** ^**a**^	**95% CI**
Diagnosis match^b^						
Acne, rosacea, and hidradenitis (*n* = 39)	0.95	0.88, 1.01	0.96	0.91, 1.01	0.98	0.94, 1.01
Papulosquamous rashes (*n* = 50)	0.74	0.61, 0.86	0.81	0.70, 0.91	0.83	0.73, 0.93
Malignant neoplasm (*n* = 25)	0.76	0.53, 0.99	0.90	0.77, 1.02	0.86	0.69, 1.02
Treatment match^b^						
Biopsy match for malignancies^c^ (*n* = 17)	0.82	0.61, 1.03	0.82	0.62, 1.02	0.93	0.81, 1.05

^a^Means represent margin values estimated using mixed models accounting for repeated assessments across modes (i.e., repeated patient subjects), with modes of evaluation as predictors.

^b^Means of percent matches (ranging from 0 to 1).

^c^Subset of matched malignancy and clinical treatment being biopsied.

**Table 3:** Extent of agreement in diagnosis and treatment match, as well as related medical outcomes, across the three assessment modes and raters.

**Categorical outcomes**	**OR**	**95% CI**	**p** **value**

Outcome: Diagnosis match			
Mode			
Taken by resident	Ref.		
Patient, unassisted	0.59	0.45, 0.79	< 0.001⁣^∗^
Patient, assisted	0.83	0.62, 1.11	0.20
Raters			
TD	Ref.		
Resident	0.66	0.52, 0.83	< 0.001⁣^∗^
Outcome: Treatment match			
Mode			
Taken by resident	Ref.		
Patient, unassisted	0.76	0.59, 0.97	0.03⁣^∗^
Patient, assisted	0.98	0.76, 1.25	0.85
Raters			
TD	Ref.		
Resident	1.35	1.10, 1.65	0.004⁣^∗^
Outcome: Asked for history			
Mode			
Taken by resident	Ref.		
Patient, unassisted	1.98	1.55, 2.82	< 0.001⁣^∗^
Patient, assisted	1.59	1.25, 2.03	< 0.001⁣^∗^
Raters			
TD	Ref.		
Resident	1.43	1.17, 1.75	< 0.001⁣^∗^

**Continuous outcomes**	**Coefficient** **(** **β** **)**	**95% CI**	**p** **value**

Outcome: Mean follow-up			
Mode			
Taken by resident	Ref.		
Patient, unassisted	−4.21	−7.45, −0.97	0.01⁣^∗^
Patient, assisted	−1.52	−4.76, 1.71	0.36
Raters			
TD	Ref.		
Resident	0.49	−2.16, 3.13	0.72
Outcome: Time to diagnosis			
Mode			
Taken by resident	Ref.		
Patient, unassisted	2.51	1.20, 3.82	< 0.01⁣^∗^
Patient, assisted	0.74	−0.57, 2.05	0.27
Raters			
TD	Ref.		
Resident	−5.42	−6.49, −4.35	< 0.001⁣^∗^
Outcome: Confidence			
Mode			
Taken by resident	Ref.		
Patient, unassisted	−0.48	−0.70, −0.26	< 0.001⁣^∗^
Patient, assisted	−0.17	−0.39, 0.04	0.12
Raters			
TD	Ref.		
Resident	−1.49	−1.66, −1.31	< 0.001⁣^∗^

Abbreviation: TD, attending physician.

⁣^∗^Significant at *p* value < 0.05.

**Table 4:** Extent of agreement in diagnosis and treatment match, as well as related medical outcomes, across the three assessment modes, medical provider roles, and patient's education.

**Categorical outcomes**	**OR**	**95% CI**	**p** **value**

Outcome: Diagnosis match			
Mode			
Taken by resident	Ref.		
Patient, unassisted	0.59	0.45, 0.79	< 0.001⁣^∗^
Patient, assisted	0.83	0.62, 1.11	0.204
Raters			
TD	Ref.		
Resident	0.66	0.52, 0.83	< 0.001⁣^∗^
Education			
University degree	Ref.		
No university degree	0.71	0.44, 1.15	0.166
Outcome: Treatment match			
Mode			
Taken by resident	Ref.		
Patient, unassisted	0.76	0.59, 0.97	0.029⁣^∗^
Patient, assisted	0.98	0.76, 1.25	0.851
Raters			
TD	Ref.		
Resident	1.35	1.10, 1.65	0.004⁣^∗^
Education			
University degree	Ref.		
No university degree	1.15	0.76, 1.75	0.504
Outcome: Asked for history			
Mode			
Taken by resident	Ref.		
Patient, unassisted	1.97	1.54, 2.52	< 0.001⁣^∗^
Patient, assisted	1.59	1.24, 2.02	< 0.001⁣^∗^
TD/R			
Attendings	Ref.		
Resident	1.43	1.18, 1.75	< 0.001⁣^∗^
Education			
University degree	Ref.		
No university degree	1.33	0.90, 1.98	0.154

**Continuous outcomes**	**Coefficient (** **β** **)**	**95% CI**	**p** **value**

Outcome: Mean follow-up			
Mode			
Taken by resident	Ref.		
Patient, unassisted	−4.20	−7.45, −0.96	0.011⁣^∗^
Patient, assisted	−1.51	−4.76, 1.74	0.363
Raters			
TD	Ref.		
Resident	0.48	−2.18, 3.13	0.725
Education			
University degree	Ref.		
No university degree	−2.49	−7.38, 2.40	0.319
Outcome: Time to diagnosis			
Mode			
Taken by resident	Ref.		
Patient, unassisted	2.49	1.18, 3.80	< 0.001⁣^∗^
Patient, assisted	0.73	−0.58, 2.04	0.276
Raters			
TD	Ref.		
Resident	−5.40	−6.48, −4.32	< 0.001⁣^∗^
Education			
University degree	Ref.		
No university degree	1.63	−0.52, 3.77	0.137
Outcome: Confidence			
Mode			
Taken by resident	Ref.		
Patient, unassisted	−0.47	−0.69, −0.25	< 0.001⁣^∗^
Patient, assisted	−0.18	−0.40, 0.04	0.107
Raters			
TD	Ref.		
Resident	−1.48	−1.66, −1.30	< 0.001⁣^∗^
Education			
University degree	Ref.		
No university degree	−0.29	−0.66, 0.08	0.126

Abbreviation: TD, attending physician.

⁣^∗^Significant at *p* value < 0.05.

## Data Availability

The data are available upon request from the corresponding author.

## Supporting Information

Supporting Information Additional supporting information can be found online in the Supporting Information section. The supporting information includes additional methodological details, extended data analyses, and Tables S1–S3 that provide further insights into the experimental results. While Forms S3 and S4 contain raw data used for statistical analysis, Tables S4 and S5 report the interrater agreement in diagnosis and treatment concordance, as well as related medical outcomes, across the three assessment modes and accounting for interaction effects between modes and raters. These materials enhance the reproducibility of our findings and provide additional context for the results discussed in the main manuscript.
