# Supplementary material for: Teledermatology Diagnostic Accuracy: A Randomized Cohort Study Comparing Three Image Acquisition Techniques
Source: Int J Telemed Appl. 2025 Sep 24;2025:5789165. doi: 10.1155/ijta/5789165 (PMC12488292; doi:10.1155/ijta/5789165)
Supplement: Supporting Information — Additional supporting information can be found online in the Supporting Information section. The supporting information includes additional methodological details, extended data analyses, and Tables S1–S3 that provide further insights into the experimental results. While Forms S3 and S4 contain raw data used for statistical analysis, Tables S4 and S5 report the interrater agreement in diagnosis and treatment concordance, as well as related medical outcomes, across the three assessment modes and accounting for interaction effects between modes and raters. These materials enhance the reproducibility of our findings and provide additional context for the results discussed in the main manuscript. [file 5789165.f1.zip › Form 3 20-08-03.pdf]

Form 3

To be filled by the researcher during the teledermatology session

	Dermatologist 3:	Dermatologist 4:	Resident 2:
Diagnosis			
Therapy	☐ Topical steroid<br>☐ Topical antibiotic<br>☐ Topical antifungal<br>☐ Topical combination (specify)<br>☐ Systemic steroid or immunosuppressant<br>☐ Systemic antibiotic<br>☐ Systemic antifungal<br>☐ Observation<br>☐ Other:	☐ Topical steroid<br>☐ Topical antibiotic<br>☐ Topical antifungal<br>☐ Topical combination (specify)<br>☐ Systemic steroid or immunosuppressant<br>☐ Systemic antibiotic<br>☐ Systemic antifungal<br>☐ Observation<br>☐ Other:	☐ Topical steroid<br>☐ Topical antibiotic<br>☐ Topical antifungal<br>☐ Topical combination (specify)<br>☐ Systemic steroid or immunosuppressant<br>☐ Systemic antibiotic<br>☐ Systemic antifungal<br>☐ Observation<br>☐ Other:
Follow Up:			
Time to dx:			
Confidence:			

Institutional Review Board
American University of Beirut
12 AUG 2020
APPROVED
