# Supplementary material for: Teledermatology Diagnostic Accuracy: A Randomized Cohort Study Comparing Three Image Acquisition Techniques
Source: Int J Telemed Appl. 2025 Sep 24;2025:5789165. doi: 10.1155/ijta/5789165 (PMC12488292; doi:10.1155/ijta/5789165)
Supplement: Supporting Information — Additional supporting information can be found online in the Supporting Information section. The supporting information includes additional methodological details, extended data analyses, and Tables S1–S3 that provide further insights into the experimental results. While Forms S3 and S4 contain raw data used for statistical analysis, Tables S4 and S5 report the interrater agreement in diagnosis and treatment concordance, as well as related medical outcomes, across the three assessment modes and accounting for interaction effects between modes and raters. These materials enhance the reproducibility of our findings and provide additional context for the results discussed in the main manuscript. [file 5789165.f1.zip › Form 4 20-08-03.pdf]

Form 4: To be used by the physician when guiding the patient on taking a photo

- ☐ Ensure there is adequate lighting
- ☐ Eliminate all distractors such as jewelry
- ☐ Make sure that the lesion is surrounded by a white background
- ☐ Make sure the photo is in focus by tapping on the screen
- ☐ Take a photo of the lesion from far to be able to determine the location of the lesion and take a photo of the lesion up-close.
- ☐ Check the photo to make sure it is not blurry, if it is retake the photo

O'Connor DM, Jew OS, Perman MJ, Castelo-Soccio LA, Winston FK, McMahon PJ. Diagnostic Accuracy of Pediatric Teledermatology Using Parent-Submitted Photographs: A Randomized Clinical Trial. *JAMA Dermatol.* 2017;153(12):1243–1248. doi:10.1001/jamadermatol.2017.4280
Muraco L. Improved Medical Photography: Key Tips for Creating Images of Lasting Value [published online ahead of print, 2020 Jan 2]. *JAMA Dermatol.* 2020;10.1001/jamadermatol.2019.3849. doi:10.1001/jamadermatol.2019.3849

Institutional Review Board
American University of Beirut
12 AUG 2020
APPROVED
