# Supplementary material for: Teledermatology Diagnostic Accuracy: A Randomized Cohort Study Comparing Three Image Acquisition Techniques
Source: Int J Telemed Appl. 2025 Sep 24;2025:5789165. doi: 10.1155/ijta/5789165 (PMC12488292; doi:10.1155/ijta/5789165)
Supplement: Supporting Information — Additional supporting information can be found online in the Supporting Information section. The supporting information includes additional methodological details, extended data analyses, and Tables S1–S3 that provide further insights into the experimental results. While Forms S3 and S4 contain raw data used for statistical analysis, Tables S4 and S5 report the interrater agreement in diagnosis and treatment concordance, as well as related medical outcomes, across the three assessment modes and accounting for interaction effects between modes and raters. These materials enhance the reproducibility of our findings and provide additional context for the results discussed in the main manuscript. [file 5789165.f1.zip › Supplementary Tables.TD.docx]

**Supplementary Tables**

**Table S1.** Extent of agreement in diagnosis and treatment match, as well as related medical outcomes, across the three assessment modes, **among attendings**

|  | Pictures taken by resident | Pictures taken by patient without assistance | 95% CI | p-value | Pictures taken by patient with assistance | 95% CI | p-value | Estat ICC |
| --- | --- | --- | --- | --- | --- | --- | --- | --- |
| Categorical variables | **Odds Ratio (OR)** |  |  |  |  |  |  |  |
| Diagnosis match | Ref=1 | 0.54 | 0.356, 0.822 | 0.004* | 0.789 | 0.514, 1.21 | 0.278 | 0.372 [0.262, 0.497] |
| Treatment match | Ref=1 | 0.786 | 0.553, 1.12 | 0.180 | 0.852 | 0.600, 1.21 | 0.372 | 0.376 [0.281, 0.482] |
| Asked for history | Ref=1 | 2.09 | 1.48, 2.95 | <0.001* | 1.51 | 1.08, 2.12 | 0.017* | 0.303 [0.212, 0.411] |
| Continuous variables | **Coefficient (β)** |  |  |  |  |  |  |  |
| Mean follow-up *(in days)* | Ref=0 | -6.56 | -12.10, -1.03 | 0.020* | -5.62 | -11.2, -0.088 | 0.046* | 0.125 [0.072, 0.207] |
| Time to diagnosis *(in days)* | Ref=0 | 4.12 | 1.89, 6.35 | <0.001* | 1.26 | -0.969, 3.49 | 0.268 | 0.208 [0.148, 0.284] |
| Confidence | Ref=0 | -0.437 | -0.76, -0.114 | 0.008* | -0.280 | -0.60, 0.044 | 0.090 | 0.156 [0.100, 0.235] |

*Significant at p-value <0.05

**Table S2.** Extent of agreement in diagnosis and treatment match, as well as related medical outcomes, across the three assessment modes, **among residents**

|  | Pictures taken by resident | Pictures taken by patient without assistance | 95% CI | p-value | Pictures taken by patient with assistance | 95% CI | p-value | Estat ICC |
| --- | --- | --- | --- | --- | --- | --- | --- | --- |
| Categorical variables | **Odds Ratio (OR)** |  |  |  |  |  |  |  |
| Diagnosis match | Ref=1 | 0.653 | 0.438, 0.972 | 0.036* | 0.863 | 0.576, 1.29 | 0.472 | 0.439 [0.333, 0.550] |
| Treatment match | Ref=1 | 0.718 | 0.501, 1.03 | 0.070 | 1.12 | 0.786, 1.60 | 0.526 | 0.427 [0.331, 0.529] |
| Asked for history | Ref=1 | 1.92 | 1.34, 2.74 | <0.001* | 1.73 | 1.21, 2.48 | 0.003* | 0.387 [0.290, 0.493] |
| Continuous variables | **Coefficient (β)** |  |  |  |  |  |  |  |
| Mean follow-up *(in days)* | Ref=0 | -1.86 | -5.19, 1.47 | 0.274 | 2.58 | -0.759, 5.91 | 0.130 | 0.334 [0.270, 0.404] |
| Time to diagnosis *(in days)* | Ref=0 | 0.909 | -0.441, 2.26 | 0.187 | 0.211 | -1.14, 1.56 | 0.760 | 0.312 [0.249, 0.384] |
| Confidence | Ref=0 | -0.521 | -0.815, -0.228 | <0.001* | -0.066 | -0.359, 0.228 | 0.661 | 0.344 [0.281, 0.414] |

*Significant at p-value <0.05

**Table S3.** Match in diagnosis and treatment, and related outcomes across the three assessment modes **(N=351)**

|  | Pictures taken by patient without assistance | | Pictures taken by patient with assistance | | | Pictures taken by resident | |
| --- | --- | --- | --- | --- | --- | --- | --- |
|  | **TD1** | **R1** | **TD2** | **R2** | | **TD3** | **R3** |
| Diagnosis match, n (%) | 261 (74.4) | 245 (69.8) | 279 (79.5) | 259 (73.8) | | 289 (82.3) | 266 (75.8) |
| Acne, Rosacea and Hidradenitis diagnosis match | 34 (87.2) | 33 (84.6) | 37 (94.9) | 32 (82.1) | | 36 (92.3) | 36 (92.3) |
| Malignant neoplasm diagnosis match | 18 (72.0) | 15 (60.0) | 19 (76.0) | 20 (80.0) | | 19 (76.0) | 18 (72.0) |
| Treatment match, n (%) | 139 (39.6) | 149 (42.4) | 144 (41) | 176 (50.1) | | 154 (43.9) | 168 (47.9) |
| Biopsy match for malignancies | 11 (64.7) | 11 (64.7) | 13 (76.5) | 13 (76.5) | | 12 (70.6) | 13 (76.5) |
| Asked for history, n (%) | 206 (58.7) | 184 (52.4) | 156 (44.4) | 220 (62.7) | 215 (61.2) | | 179 (51) |
| Mean follow-up *(in days)*, mean ±SD | 15.7 **±**33.8 | 16.6 **±**22.0 | 16.7 **±**30.5 | 21.1 **±**35.7 | | 22.3 **±**52.3 | 18.5 **±**23.1 |
| Time to diagnosis *(in days)*, mean ±SD | 22.4 **±**18.3 | 15.2 **±**10.8 | 19.5 **±**16.3 | 14.5 **±**10.9 | | 18.3 **±**16.2 | 14.3 **±**11.3 |
| Confidence, mean ±SD | 7.2 **±**2.5 | 5.6 **±**2.5 | 7.4 **±**2.4 | 6.1 **±**2.5 | | 7.7 **±**2.3 | 6.1 **±**2.3 |

**TD:** Attending physician

**R:** Resident

**Table S4.** Inter-rater agreement in diagnosis match and related medical outcomes between raters across the three assessment modes

|  | Pictures taken by patient without assistance | | | Pictures taken by patient with assistance | | | Pictures taken by resident | | |
| --- | --- | --- | --- | --- | --- | --- | --- | --- | --- |
| Categorical variables | **TD1** | **R1** | **κ** **(95% CI)** | **TD2** | **R2** | κ **(95% CI)** | **TD3** | **R3** | **κ** **(95% CI)** |
| Diagnosis match | 261 (74.36) | 245 (69.8) | 0.308 (0.200, 0.416) | 279 (79.49) | 259 (73.79) | 0.255 (0.142, 0.369) | 289 (82.34) | 266 (75.78) | 0.342 (0.226, 0.457) |
| Treatment match | 139 (39.6) | 149 (42.4) | 0.294 (0.193 - 0.395) | 144 (41) | 176 (50.1) | 0.237 (0.137 - 0.337) | 154 (43.9) | 168 (47.9) | 0.206 (0.104 - 0.309) |
| Asked for history | 206 (58.7) | 184 (52.4) | 0.209 (0.106 - 0.313 | 156 (44.4) | 220 (62.7) | 0.291 (0.193 - 0.390) | 215 (61.2) | 179 (51) | 0.169 (0.067 - 0.272) |
| Continuous variables | **TD1** | **R1** | **ICC (95% CI)** | **TD2** | **R2** | **ICC (95% CI)** | **TD3** | **R3** | **ICC (95% CI)** |
| Mean follow-up | 15.7 **±**33.8 | 16.6 **±**22.0 | 0.158 (0.054, 0.258) | 16.7 ±30.5 | 21.1 **±**35.7 | 0.214 (0.112, 0.311) | 22.3 **±**52.3 | 18.5 **±**23.1 | 0.075 (-0.029, 0.178) |
| Time to diagnosis | 22.4 **±**18.3 | 15.2 **±**10.8 | 0.270 (0.148, 0.379) | 19.5 ±16.3 | 14.5 **±**10.9 | 0.226 (0.123, 0.325) | 18.3 **±**16.2 | 14.3 **±**11.3 | 0.245 (0.145, 0.341) |
| Confidence | 7.2 **±**2.5 | 5.6 **±**2.5 | 0.323 (0.131, 0.475) | 7.4 ±2.4 | 6.1 **±**2.5 | 0.229 (0.106, 0.342) | 7.7 **±**2.3 | 6.1 **±**2.3 | 0.247 (0.090, 0.383) |

**Κ:** Kappa

**ICC:** intraclass correlation coefficients

**TD**: attending physician

**R:** Resident

**Table S5**. Extent of agreement in diagnosis and treatment match, as well as related medical outcomes, with an interaction term between assessment of modes and raters

| **Categorical Outcomes** | **OR** | **95% CI** | **P-VALUE** |
| --- | --- | --- | --- |
| Outcome: Diagnosis match | | | |
| **Mode** |  |  |  |
| Taken by resident | Ref. |  |  |
| Patient, unassisted | 0.53 | 0.35, 0.81 | 0.003* |
| Patient, assisted | 0.78 | 0.51, 1.21 | 0.27 |
| **Raters** |  |  |  |
| TD | Ref. |  |  |
| Resident | 0.59 | 0.38, 0.90 | 0.01* |
| **Mode*Raters** |  |  |  |
| Patient unassisted*R | 1.25 | 0.70, 2.22 | 0.45 |
| Patient assisted*R | 1.10 | 0.61, 1.99 | 0.74 |
| Outcome: Treatment match | | | |
| **Mode** |  |  |  |
| Taken by resident | Ref. |  |  |
| Patient, unassisted | 0.79 | 0.56, 1.12 | 0.18 |
| Patient, assisted | 0.85 | 0.60, 1.21 | 0.38 |
| **Raters** |  |  |  |
| TD | Ref. |  |  |
| Resident | 1.26 | 0.89, 1.78 | 0.19 |
| **Mode*Raters** |  |  |  |
| Patient unassisted*R | 0.93 | 0.56, 1.52 | 0.77 |
| Patient assisted*R | 1.30 | 0.80, 2.13 | 0.29 |
| Outcome: Asked for History | | | |
| **Mode** |  |  |  |
| Taken by resident | Ref. |  |  |
| Patient, unassisted | 2.12 | 1.50, 2.98 | <0.001* |
| Patient, assisted | 1.52 | 1.08, 2.13 | 0.02* |
| **Raters** |  |  |  |
| TD | Ref. |  |  |
| Resident | 1.45 | 1.03, 2.04 | 0.03* |
| **Mode*Raters** |  |  |  |
| Patient unassisted*R | 0.87 | 0.54, 1.42 | 0.58 |
| Patient assisted*R | 1.11 | 0.68, 1.79 | 0.68 |
| **Continuous outcomes** | **Coefficient (β)** | **95% CI** | **P-VALUE** |
| Outcome: Mean follow-up |  |  |  |
| **Mode** |  |  |  |
| Taken by resident | Ref. |  |  |
| Patient, unassisted | -6.56 | -11.13, -1.99 | 0.005* |
| Patient, assisted | -5.62 | -10.19, -1.05 | 0.02* |
| **Raters** |  |  |  |
| TD | Ref. |  |  |
| Resident | -3.81 | -8.39, 0.76 | 0.10 |
| **Mode*Raters** |  |  |  |
| Patient unassisted*R | 4.70 | -1.76, 11.17 | 0.15 |
| Patient assisted*R | 8.20 | 1.74, 14.66 | 0.01* |
| Outcome: Time to diagnosis |  |  |  |
| **Mode** |  |  |  |
| Taken by resident | Ref. |  |  |
| Patient, unassisted | 4.12 | 2.27, 5.97 | <0.001* |
| Patient, assisted | 1.26 | -0.59, 3.11 | 0.18 |
| **Raters** |  |  |  |
| TD | Ref. |  |  |
| Resident | -4.00 | -5.85, -2.15 | <0.001* |
| **Mode* Raters** |  |  |  |
| Patient unassisted*R | -3.21 | -5.82, -0.60 | 0.02* |
| Patient assisted*R | -1.05 | -3.67, 1.56 | 0.43 |
| Outcome: Confidence |  |  |  |
| **Mode** |  |  |  |
| Taken by resident | Ref. |  |  |
| Patient, unassisted | -0.44 | -0.74, -0.13 | 0.005* |
| Patient, assisted | -0.28 | -0.59, 0.03 | 0.08 |
| **Raters** |  |  |  |
| TD | Ref. |  |  |
| Resident | -1.53 | -1.84, -1.22 | <0.001* |
| **Mode*Raters** |  |  |  |
| Patient unassisted*R | -0.08 | -0.52, 0.35 | 0.70 |
| Patient assisted*R | 0.21 | -0.22, 0.65 | 0.34 |

*Significant at p-value<0.05

**TD:** Attending physician

**R:** Resident
